# Utilization of a Mobile Multifunctional Workstation for Coronavirus Nasopharyngeal/Oropharyngeal Specimen Collection

**DOI:** 10.3389/fpubh.2021.794359

**Published:** 2022-01-24

**Authors:** Xiaojing Lian, Lili Zhang, Yang Zhao, Yuhua Li, Xuemin Jing, Xin Liu, Jianing Han, Jianhong Ma, Yongyong Zhang, Huimin Guo, Xiaojie Huang

**Affiliations:** ^1^Integrated Traditional Chinese and Western Medicine Center, Beijing Youan Hospital, Capital Medical University, Beijing, China; ^2^Nursing Department of Beijing Youan Hospital, Capital Medical University, Beijing, China; ^3^Department of Dermatology, Shunyi Maternal and Children's Hospital of Beijing Children's Hospital, Beijing, China; ^4^Second Department of Infection, Beijing Youan Hospital, Capital Medical University, Beijing, China; ^5^Intervention Department, Beijing Youan Hospital, Capital Medical University, Beijing, China; ^6^Department of Critical Care Medicine, Beijing Youan Hospital, Capital Medical University, Beijing, China; ^7^Department of Infection, Beijing Youan Hospital, Capital Medical University, Beijing, China; ^8^Outpatient Department, Beijing Youan Hospital, Capital Medical University, Beijing, China; ^9^Center for Infectious Disease, Beijing Youan Hospital, Capital Medical University, Beijing, China

**Keywords:** mobile multifunctional workstation, satisfaction, COVID-19, specimen collection, nasopharyngeal/oropharyngeal specimen collection

## Abstract

**Objectives:**

This study aimed to explore the utilization of a mobile multifunctional workstation for COVID-19 sample collection.

**Methods:**

Twenty-four nurses and 150 individuals who took nucleic acid tests using mobile multifunctional workstations in Beijing Youan Hospital, Capital Medical University, from September to November 2020, were enrolled in the study as the observation group. As the control group, we included 36 nurses and 150 individuals who did not use the workstations from June to September 2020. We compared the two groups on (1) comfort of working environment, self-perceived security, the convenience of information system, operational process flexibility, pharyngeal acquisition visibility, and effectiveness of communication among nurses; and (2) self-perceived safety, waiting time, and overall satisfaction among individuals who took nucleic acid tests.

**Results:**

The satisfaction score of nurses in the observation group of nurses were significantly higher than those of the control group (OR = 17.297 95% CI:4.294, 69.673), as well as the convenience of the information system (OR = 6.250 95% CI: 1.775, 22.008), and communication effectiveness (OR = 5.588 95% CI: 1.590, 19.646). Among individuals who took nucleic acid tests, the overall satisfaction (*P* < 0.05) and self-perceived security (*P* < 0.05) had statistical differences between the observation group and the control group.

**Conclusions:**

The mobile multifunctional workstation for specimen collection could improve the comfort of the working environment, the convenience of information systems, and the effectiveness of communication among nurses.It can improve satisfaction and self-perceived security among people who took nucleic acid tests.

## Introduction

The transmission of coronavirus (COVID-19) takes place mainly through respiratory droplets and close contact. If people are exposed to aerosols in a closed environment, it carries an increased infection risk (1). Taking nasopharyngeal swabs for nucleic acid detection has become a convenient screening method (2). When collecting samples, contact with respiratory exhalations or secretions of patients with COVID-19 is a high-risk procedure. Thus, the *Guidelines for the Use of Common Personal Protective Equipment in the Prevention and Control of SARS-CoV-2* (3) stipulates that in case of close contact with a patient who has confirmed or suspected COVID-19, collectors must comply with the third-level protection standards to ensure the safety of healthcare workers collecting specimens.

However, complying with third-level protection causes considerable discomfort. In addition, the costs of PPE and the amount of medical waste produced is very high. Moreover, specimen collection, processing, and storage should follow the procedure of early collection, aseptic operation, and cryopreservation (4). However, samples are usually placed at room temperature after collection, which does not meet the sample preservation regulations, thus affecting the test accuracy. The study aimed to explore the application of mobile multifunctional workstations for sample collection.

The workstation is composed of a collection kiosk and a multifunction console. We creatively integrated an information system into the workstation, which solved the current issue of workstations not having information systems. The information system enables nurses to complete registration by scanning QR codes efficiently, and medical staff can easily trace the sample throughout the process. The original workstation generally had a closed negative- or positive-pressure design, with built-in air conditioning. We adopted a closed positive-pressure design and incorporated a fresh air system—the cleanliness reached the ISO6 cleanroom standard. Based on traditional ultraviolet disinfection, automatic disinfection outside the workstation was added. Furthermore, we also added an anti-fog panel, inductive dustbin, constant temperature refrigerator, and other details that improved the practicability and convenience of use of the workstations.

## Methods

### Ethical Approval

The study was conducted in compliance with the Declaration of Helsinki. All the participants signed informed consent. The studies involving human participants were reviewed and approved by the Ethics Committee of Beijing Youan Hospital Affiliated with the Capital Medical University.

### Study Population

Twenty-four nurses from Beijing Youan Hospital working from September to November 2020 in the screening outpatient department were selected as the observation group. They used the mobile multifunctional sample collection kiosk to collect throat swabs from individuals who took nucleic acid tests. From June to September 2020, 36 nurses from the outside collection team of the nucleic acid testing group served as the control group. They followed third-level protection standards while collecting throat swabs from sampled people outdoors. The selected people were all women. Furthermore, 150 individuals who took nucleic acid tests s through a collection kiosk from September to November 2020 were selected as the observation group. From June to September 2020, 150 individuals who took nucleic acid tests were selected as the observation group without being collected through the collection kiosk.

The observation group nurses wore nurse uniforms, medical-surgical masks, and disposable gloves in the collection pavilion. They are equipped with a head-mounted LED light supplement device to perform throat swab sampling. Then, the nurses inserted their hands into the external connection gloves. After disinfection, the sampling tube is delivered from the person sampled to the nurse through the voice interaction system. After sampling, the nurse puts the throat swab into the sampling tube, seals the cap, puts it in the delivery port, and pushes the complete button to finish the sampling process. The test tube racks are placed in a row and placed in the refrigerator. After the operation, the nurse uses the foot-operated spray disinfection system to ensure the disinfection of each individual.

We distributed 60 nurse and 300 subject questionnaires. The content of the nurses' satisfaction evaluation of different work modes included the following six aspects: work environment comfort, self-perceived security, information system convenience, operating process flexibility, pharynx collection visibility, and nurse-patient communication effectiveness. The content of the collected personnel's satisfaction evaluation included the following three aspects: self-perceived security, sampling waiting time, and overall satisfaction. Both questionnaires use a 5-point Likert scale, with “very dissatisfied,” “unsatisfied,” “average,” “satisfied,” and “very satisfied” in the order from 1 to 5.

### Statistical Analysis

We analyzed the count data and normally distributed measurement data using a chi-squared or a Fisher's exact test and *t*-test separately in SPSS 25. In addition, we analyzed non-normally distributed measurement data and ordered count data by a rank-sum test of two independent samples. According to the nurses' feelings of taking nasopharyngeal swab samples, which included comfort of working environment, self-perceived security, the convenience of information system, operational process flexibility, pharyngeal acquisition visibility, and effectiveness of communication, 4–5 points were regarded as satisfaction and 1–3 points were regarded as dissatisfaction. Then the effects of age, years of work, positional titles, educational degree, and method of coronavirus nasopharyngeal/oropharyngeal specimen collection (collected through the collection kiosk or wearing PPE) on the nurses' feelings were analyzed. We carried out bivariate logistic regression analyses in SPSS 25. Then we selected the factors with a *p*-value < 0.2 for multivariate analysis.

## Results

We received 60 valid responses from the nurses. The effective recovery rate was 100.00%. Three hundred valid questionnaires were collected from the people comprising the sample. The effective recovery rate was 100%.

### Comparison of Satisfaction Scores Between the Two Groups of Nurses With Different Throat Swab Collection Working Modes

The two nursing staff groups had no statistically significant differences in basic characteristics of demography, which included working years, age, professional title, and educational background (*P* > 0.05), which means they were baseline comparable. The comfort of the nursing staff using the collection booth, self-perceived security, the convenience of the information system, the flexibility of the operation process, and the effectiveness of nurse-patient communication were statistically significant between the two groups of nurses (Table 1).

**Table 1:** Comparison of the two groups of nurses with different work patterns.

**Item**	**Control group** **(*n* = 36)**	**Observation group** **(*n* = 24)**	**Statistics**	***P*-value**
Age	36.50 (33.25–43.50)	36.50 (31.00–43.00)	*Z* = −0.280	0.780
Years of work	16.00 (10.00–23.50)	14.50 (10.00–24.00)	*Z* = −0.060	0.952
Positional titles			*X*^2^ = 0.262	0.609
Primary nurses	16	10		
Supervisor and deputy chief nurse	20	14		
Education degree junior college education			*X*^2^ = 0.045	0.832
Bachelor degree or above	7	6		
	26	18		
Comfort of working environment	2.00 (1.00–3.75)	5.00 (4.00–5.00)	*Z* = −5.449	<0.001
Self-perceived security	4.00 (3.00–5.00)	5.00 (4.00–5.00)	*Z* = −2.123	0.034
The convenience of information system	3.00 (3.00–4.00)	4.00 (4.00–4.00)	*Z* = −3.256	0.001
Operational process flexibility	0.00 (0.00–1.00)	3.00 (2.00–4.00)	*Z* = −2.601	0.009
Pharyngeal acquisition visibility	4.00 (3.00–4.00)	4.00 (4.00–4.00)	*Z* = −1.651	0.099
Effectiveness of communication	3.00 (3.00–4.00)	4.00 (4.00–5.00)	*Z* = −3.177	0.001

According to the results of bivariate and multivariate analysis, utilization of a mobile multifunctional workstation for coronavirus nasopharyngeal/oropharyngeal specimen collection can significantly improve the comfort of the working environment (OR = 17.297 95% CI: 4.294, 69.673), the convenience of information systems (OR = 6.250 95% CI: 1.775, 22.008), and effectiveness of communication (OR = 5.588 95% CI: 1.590, 19.646). Age, years of work, positional titles, educational degree, and method of coronavirus nasopharyngeal/oropharyngeal specimen collection had no statistically significant effect on operational process flexibility (*P* > 0.05). We got the same result on self-perceived security and pharyngeal acquisition visibility (Tables 2, 3).

**Table 2:** Bivariate and multivariate analysis of comfort of working environment.

	**Unadjusted odds ratio (95% CI)**	***P*-value**	**Adjusted odds ratio (95% CI)**	***P*-value**
**Age**				
18–29	Ref.	0.287	0.366(0.072,1.855)	0.225
30–39	4.200 (0.698, 25.264)	0.117		
≥40	1.213 (0.404, 3.645)	0.730		
**Years of work**				
≤ 10	Ref.	0.420	1.251 (0.305, 5.130)	0.756
10–20	1.833 (0.472, 7.216)	0.382		
≥21	2.333 (0.655, 8.309)	0.191		
**Positional titles**				
Primary nurses	Ref.			
Supervisor and deputy chief nurse	1.125 (0.405, 3.126)	0.821		
**Educational degree**				
Junior college education	Ref.			
Bachelor degree or above	0.754 (0.220, 2.585)	0.654		
**Method of coronavirus nasopharyngeal/oropharyngeal specimen collection**				
PPE	Ref.			
Workstations	15.000 (4.039, 55.708)	<0.001	17.297 (4.294, 69.673)	<0.001

**Table 3:** Bivariate and multivariate analysis of the convenience of information system and effectiveness of communication.

	**The convenience of information system**		**Effectiveness of communication**	
	**Unadjusted odds ratio (95% CI)**	***P*-value**	**Unadjusted odds ratio (95% CI)**	***P*-value**
**Age**				
18–29	Ref.	0.502	Ref.	0.449
30–39	0.846 (0.171, 4.198)	0.838	1.500 (0.245, 9.179)	0.661
≥40	1.786 (0.578, 5.522)	0.314	0.339 (0.577, 0.187)	0.339
**Years of work**				
≤ 10	Ref.	0.561	Ref.	0.636
10–20	1.111 (0.294, 4.205)	0.877	0.655 (0.160, 2.680)	0.556
≥21	1.889 (0.530, 6.727)	0.326	0.530 (0.143, 1.962)	0.342
**Positional titles**				
Primary nurses	Ref.		Ref.	
Supervisor and deputy chief nurse	0.893 (0.314, 2.537)	0.832	1.792 (0.625, 5.142)	0.278
**Educational degree**				
Junior college education	Ref.		Ref.	
Bachelor degree or above	1.381 (0.400, 4.767)	0.610	0.655 (0.176, 2.437)	0.528
**Method of coronavirus nasopharyngeal/oropharyngeal specimen collection**				
PPE	Ref.		Ref.	
Workstations	6.250 (1.775, 22.008)	0.004	5.588 (1.590, 19.646)	0.007

### Comparison of Satisfaction Scores Between the Two Groups Among Individuals Who Were Collected Throat Specimens Before and After Using the Collection Kiosk

There were no statistically significant differences in terms of sex, age, and educational level between the two groups (*P* > 0.05), which means they were comparable. The scores of self-perceived safety and overall satisfaction during the observation group's throat swab sampling process were higher than those of the control group, and the difference (*P* < 0.05) was statistically significant (Table 4).

**Table 4:** Comparison between two groups of people requiring nasopharyngeal swabs.

**Group**	**Control group (*n* = 150)**	**Observation group (*n* = 150)**	***Z*-value**	***P*-value**
Sex			2.940	0.086
Male	107	93		
Female	43	57		
Age	43.00 (31.50–52.00)	41.00 (30.00–51.00)	−0.178	0.858
Education degree	3.50 (1.75–3.00)	3.00 (2.00–3.00)	−0.682	0.495
Self-perceived security	2.00 (1.75–3.00)	2.00 (2.00–3.00)	−1.965	0.049
Waiting time	3.50 (2.00–4.00)	3.00 (2.25–4.00)	−0.063	0.950
Overall satisfaction	3.00 (2.00–3.00)	3.00 (2.00–4.00)	−2.053	0.040

## Discussion

The convenience of the information system of the observation group among nurses is 6.25 times that of the control group. A significant innovation of the current work is that an information system was installed into the workstation. The kiosk supports the scanning and registering sampling of test tubes and can realize information registration and transmission through mainstream barcodes/two-dimensional codes. After scanning, data will be uploaded automatically, and the staff can view the sample information through the medical system. The information system reduces the burden of nurses in terms of writing and improves the comfort of use. It is convenient for data maintenance, unified management, and traceability. Reducing the workload is conducive to nurses undertaking more sampling work. Simultaneously, it also grants the medical system a greater sampling capacity, which is of significance for epidemic prevention and control.

During the collection of nucleic acid, wearing N95 masks and goggles for a long time causes facial indentation, dermatitis, acne, and itching (5–8). High mental pressure, being unaccustomed to and uncomfortable wearing PPE, and inconvenient movement all affect the clarity of vision, operating sensitivity, and accuracy (9–11). Especially during summer, wearing PPE for a long time causes physical exhaustion among medical staff. A series of manifestations such as insufficient blood and oxygen supply, and being prone to collapse and heatstroke further increase the difficulty and intensity of the work of nasopharyngeal swab collection (12). Further, during winter, the low temperatures prevent nurses from collecting samples outdoors for a long time. Thus, nurses are dissatisfied with the working environment.

To make the nurses more comfortable, the collection kiosk adopts a fully enclosed state with built-in air conditioning, which can prevent infection by the stable positive pressure control, and a 99% HEPA clean filter device (13). This design reduces the necessitation of using PPE. The comfort of the working environment of the observation group among nurses is 15.00 times that of the control group. It ensures that nurses are energetic and not overburdened, which provides a guarantee for long-term nucleic acid sampling and is of significance to the normalization of epidemic prevention and control.

Moreover, improving the comfort of nurses' working environment may positively affect nurses' negative emotions because some studies have shown that nurses' negative emotions, such as anxiety, are related to job dissatisfaction (14). The high prevalence of emotional distress in medical staff during the COVID-19 pandemic deserves great attention (15–19). Nurses are more prone to anxiety in the late stage of epidemic development (20–22). The relationship between the negative emotions and the comfort of the working environment is worthy of further study in the future.

Protective equipment that satisfies the protective standard usually affects the sense of hearing, which can lead to difficulties in communication between nurses and patients taking the nucleic acid tests. This collection booth has a voice interaction system to solve this problem. The satisfaction with communication among nurses in the observation group was 5.59 times that of the control group.

In the process of application, we found another advantage of the multi-functional workstation compared with the traditional PPE. The multi-functional workstation is more environmentally-friendly. Some components of personal protective equipment are produced from non-renewable fossil resources. The process of mass production of PPE will also lead to large consumption of fossil fuels (23). Because of the poor recyclability of these plastic compounds (24), improper treatment can cause plastic pollution (25, 26), thus affecting the ecological environment of the sea and soil (24, 27, 28). In addition, the virus remaining on PPE needs to be properly disinfected, otherwise, it may cause virus transmission.

The author believes that the sampling field of view of medical staff is significantly reduced under the third-level protection standard. The head-mounted LED light can help see the sampling location better, reduce the sampling time, and improve sampling accuracy. The *P*-value of the pharyngeal acquisition visibility referred to 0.088, which was between 0.05 and 0.1. It may be related to the small sample size, which needs to be expanded in the future.

The overall satisfaction and the self-perception security of the observation group among people who took nuclear acid tests were statistically different from those of the control group, which means utilization of a mobile multifunctional workstation can help to reduce people's anxiety. Those who have close contact with confirmed cases of the new COVID-19 should be tested for nucleic acid. Improving satisfaction with nucleic acid and reducing anxiety is conducive to people's cooperation in epidemic prevention and control.

Besides, we put the induction dustbin, hand disinfectant, and pharyngeal swab in the sink to expand the space and improve the operational process flexibility. But the results showed that the self-perceived security and operational process flexibility were not statistically significant between the two groups among nurses, which suggests that we should further optimize the design. In addition, sampling waiting times were not statistically significant between the two groups among people involved in throat specimen collection. It may be because that waiting time is affected by many factors such as the degree of cooperation and age of the sampled individuals. The results require further expansion of the sample size for demonstration.

One limitation of this study was that the sample size was small, so the conclusions should be treated with caution. However, through the research above, it has been preliminarily confirmed that a multifunctional collection kiosk can improve the comfort of nurses. In the future, we can further expand the sample size and conduct research across a wider range to further confirm the conclusions of this paper. Another limitation is that the nucleic acid results of the participants in our study are negative, and we cannot objectively compare the safety of the two methods. More research in this area is needed in the future.

Recently, there have been frequent outbreaks of new and severe infectious diseases worldwide, which have caused great harm to human health, such as severe acute respiratory syndrome, Ebola virus, and so on. When medical staff come into contact with such patients, they are often exposed to contaminated blood, body fluids, or aerosol environments, and the risks they face are becoming increasingly severe (29). This collection kiosk provides a safe, comfortable, convenient, efficient, and flexible working mode for medical staff. The mobile multifunctional sample collection workstation has a simple operation, which alleviates the working pressure of nurses to a certain extent, creates a comfortable and safe sampling environment for nurses and people providing the samples, and improves the overall satisfaction of both parties.

## Data Availability Statement

The raw data supporting the conclusions of this article will be made available by the authors, without undue reservation.

## Ethics Statement

The Declaration of Helsinki conducted the study. All the participants signed informed consent. The studies involving human participants were reviewed and approved by the Ethics Committee of Beijing Youan Hospital Affiliated to Capital Medical University.

## Author Contributions

HG and LZ contributed to the design of this study. JM, YL, XJ, XLiu, JH, and YZhan contributed to data collection. YZhao and XLia analyzed the data and wrote the article. All authors have read and approved the final manuscript.

## Funding

This work was supported by the Major Project of Beijing Municipal Science and Technology Committee (No. Z201100005520063), National Science and Technology Major Project of China during the 13th Five-Year Plan Period (No.2017ZX10201101), Beijing Excellent Talent Plan (No. 2018000021223ZK04), Beijing Talent Project in the New Millennium (No. 2020A35), and Beijing Hospitals Authority Clinical medicine Development of special funding support (No. XMLX202147).

## Conflict of Interest

The authors declare that the research was conducted in the absence of any commercial or financial relationships that could be construed as a potential conflict of interest.

## Publisher's Note

All claims expressed in this article are solely those of the authors and do not necessarily represent those of their affiliated organizations, or those of the publisher, the editors and the reviewers. Any product that may be evaluated in this article, or claim that may be made by its manufacturer, is not guaranteed or endorsed by the publisher.
